# Allometric disparity in rodent evolution

**DOI:** 10.1002/ece3.521

**Published:** 2013-03-05

**Authors:** Laura A B Wilson

**Affiliations:** Kyoto University Museum, Kyoto UniversityYoshida-honmachi, Sakyo-ku, Kyoto, 606-8501, Japan

**Keywords:** Allometric trajectory, cranium, diet, morphological evolution, phenotypic covariance structure, Rodentia

## Abstract

In this study, allometric trajectories for 51 rodent species, comprising equal representatives from each of the major clades (Ctenohystrica, Muroidea, Sciuridae), are compared in a multivariate morphospace (=allometric space) to quantify magnitudes of disparity in cranial growth. Variability in allometric trajectory patterns was compared to measures of adult disparity in each clade, and dietary habit among the examined species, which together encapsulated an ecomorphological breadth. Results indicate that the evolution of allometric trajectories in rodents is characterized by different features in sciurids compared with muroids and Ctenohystrica. Sciuridae was found to have a reduced magnitude of inter-trajectory change and growth patterns with less variation in allometric coefficient values among members. In contrast, a greater magnitude of difference between trajectories and an increased variation in allometric coefficient values was evident for both Ctenohystrica and muroids. Ctenohystrica and muroids achieved considerably higher adult disparities than sciurids, suggesting that conservatism in allometric trajectory modification may constrain morphological diversity in rodents. The results provide support for a role of ecology (dietary habit) in the evolution of allometric trajectories in rodents.

## Introduction

A central goal of evolutionary studies is to understand why some clades are more morphologically diverse than others (e.g., Erwin 2007; Pigliucci 2008). To address this question, differences in morphology have been assessed using phenotypic spaces constructed from quantitative measures of anatomical variability among groups of organisms. The quantitative expression of traits that are coupled over the course of development, or to achieve a certain function, can be extracted as genetic (G matrix) or phenotypic (P matrix) covariances, providing an empirical and theoretical framework to examine how phenotypic spaces are patterned.

The study of covariance matrix evolution (Olson and Miller 1958; Lande 1976, 1979) has received much attention in the last decades. From an empirical perspective, morphological trait covariances have so far been quantified for several clades, and the potential factors underlying these patterns have been explored to assess the role of covariance structure in facilitating or constraining the evolution of traits in complex systems (e.g., Ackermann and Cheverud 2000; Marroig and Cheverud 2005, 2010; Goswami 2006, 2007; Porto et al. 2009). These covariance evolution patterns have also been central to recent theoretical attempts at conceiving generalized relationships between genotype and phenotype, with the aim of conceptualizing a theory of form (e.g., Erwin 2000; Leroi 2000; Pigliucci and Kaplan 2006; Pigliucci 2008; Rice 2008; Wilson 2012).

The majority of previous studies on covariance matrix evolution have focused on the adult stage, sampling only the “endpoint” of ontogeny. Understanding how evolution proceeds in phenotypic space also requires an understanding of the evolution of development (Hall 2000; Raff 2000) and evolutionary developmental biology (evo-devo) has yielded important evidence to show how development affects phenotypic evolution through pathways that represent their own level of biological organization and evolve to a certain extent independently from the traits they pattern (e.g., Shubin et al. 1997; Wagner et al. 2000).

Developmental insights into morphospace structuring have recently enabled an appreciation of the factors that influence the evolution of development on a macroevolutionary scale (e.g., Kavanagh et al. 2007; Renvoisé et al. 2009; Adams and Nistri 2010; Wilson et al. 2012) providing a promising avenue to address fundamental issues such as why development has evolved along a specific route (Klingenberg 2010a), and how that may be generalized to explain observed morphological diversity (e.g., Salazar-Ciudad and Jernvall 2004, 2010; Salazar-Ciudad 2006). Gerber and colleagues (Gerber et al. 2007, 2008, 2011) have formalized and exemplified the use of “allometric disparity” (but see also Klingenberg and Froese 1991; Zelditch et al. 2003), essentially using the metrical framework of morphological disparity (Sneath and Sokal 1973; Foote 1997; Erwin 2007) to compare the evolution of allometric trajectories in developmental (allometric) morphospaces. Because multivariate allometry is measured using the major axis of covariance, allometric space studies are directly comparable to covariance matrix evolution results, but importantly provide the opportunity to consider how patterns of allometric disparity relate to adult morphological diversity.

**Figure 1:**
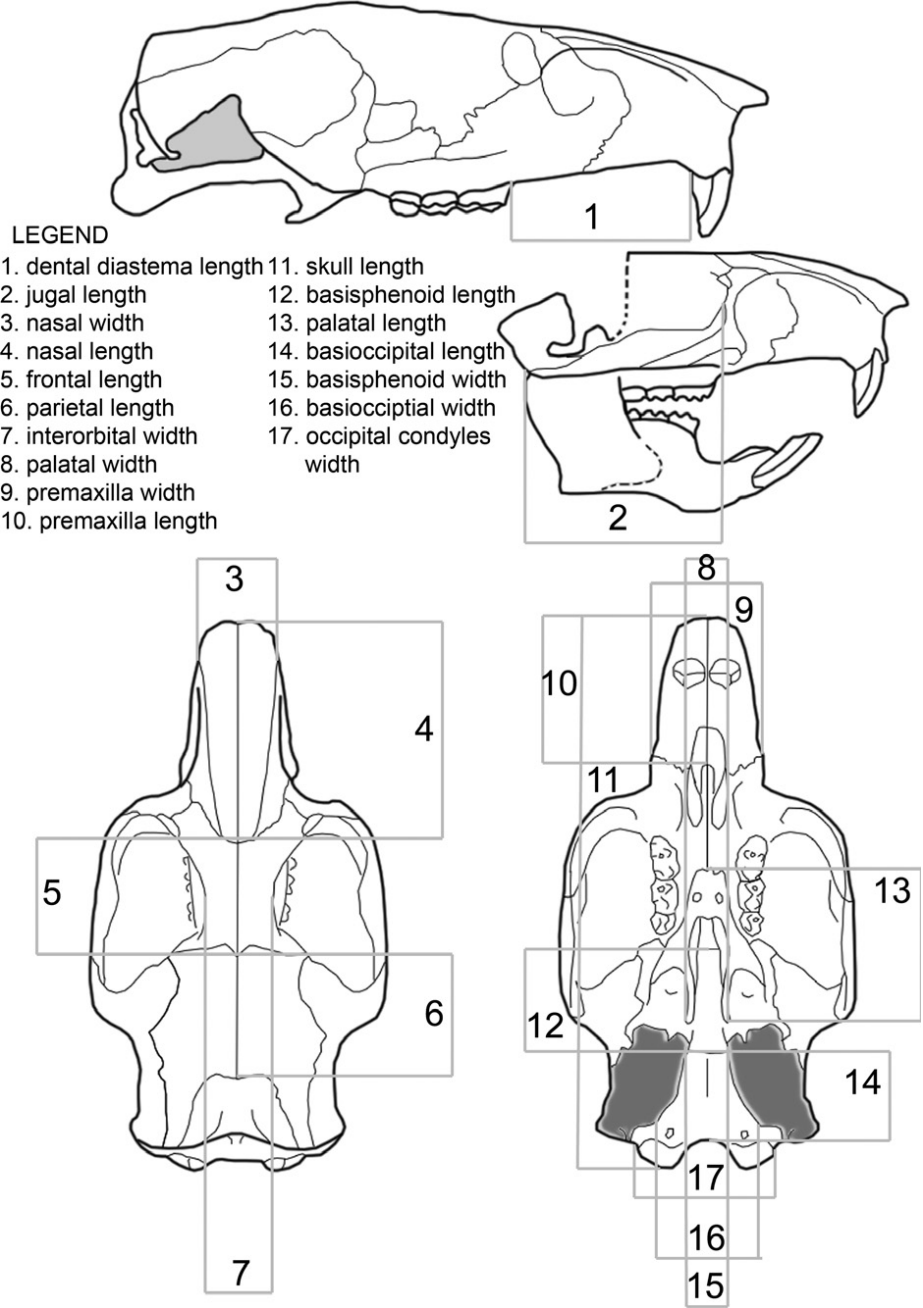
Illustration of morphometric measurements recorded on rodent crania in this study. Line drawing adapted from Carrasco and Wahlert (1999).

Wilson and Sánchez-Villagra (2010) recently performed the first exploration of allometric space for representatives from two major clades of rodents, Ctenohystrica and muroids (mice-related) (Huchon et al. 2002; Steppan et al. 2004; Blanga-Kanfi et al. 2009; Fig. 1), examining cranial growth relationships for 17 species each within each group. Changes in covariance structure were found to have occurred commonly and conspicuous differences between representatives of the two clades, such as life history strategies (altricial vs. precocial), body size variation, and locomotory habit, did not act to constrain the evolution of allometric patterns (Wilson and Sánchez-Villagra 2010). Representatives of the two clades were found to occupy overlapping portions of allometric space and a clear phylogenetic pattern was not retrieved. The addition of information on dietary habit yielded clear groupings of trajectories irrespective of phylogenetic history, indicating that ontogenetic allometries can evolve to reflect functional and ecological aspects (Klingenberg 2010b; Wilson and Sánchez-Villagra 2010).

The temporal persistence of a likely adaptive base for allometric evolution in rodents requires evaluation by examination of allometric trajectory patterns among representatives of the squirrel-related clade, which diverged from other clades early within rodent phylogeny (Huchon et al. 2002; : Steppan et al. 2004; Fabre et al. 2012). Squirrels are easily recognized, possessing distinctive morphological features, and many of these characters are considered to have remained relatively unchanged throughout the history of the crown-group (Moore 1959; Black 1963), dating back to the late Eocene (Thorington and Hoffmann 2005). Furthermore, repeated parallel evolution of giant and pygmy forms has occurred within the clade (Roth 1996). These unique features present a rich subject for comparative studies with the other major rodent lineages. Particularly: (1) Do squirrels share similar patterns of ontogenetic evolution to other rodents? (2) Are covariance structure changes common regardless of proposed skeletal conservatism and convergences? (3) Does ecology also play a role in the evolution of growth patterns in squirrels? I address these questions through the evaluation of allometric space across Rodentia, represented herein by ontogenetic series for 51 species.

## Methods

### Specimens and measurements

Landmark data were collected for specimens representing 17 species belonging to Sciuridae (Table 1). Following Wilson and Sánchez-Villagra (2010), 17 cranial measurements were derived from three-dimensional landmark coordinates collected on ontogenetic series of dry skulls using a Microscribe digitizer (Immersion Corporation, San Jose CA). Measurements were premaxilla ventral length, premaxilla width at the suture with the maxilla, palatine length, palatine width, occipital condyles width, skull length, nasal length, nasal width, frontal midline length, parietal midline length, jugal length, length of the dental diastema, interorbital width, basioccipital length, basioccipital width, basisphenoid length, and basisphenoid width (Fig. 1). In total, 507 specimens were digitized, and each ontogenetic series was represented by 30 specimens, on average (Table 1). Juvenile and subadult stages were separated based on degree of molar eruption (Anders et al. 2011) and suture closure. Stages were used only for sampling purposes to evaluate ontogenetic series across species, to achieve similar sampling for each trajectory estimate. Because suture closure pattern has not been reported for any of the species examined herein, I initially followed the method of Wilson and Sánchez-Villagra (2009) to record the sutures that closed the earliest (during growth) for specimens of *Dremomys rufigenis*, *Lariscus insignis,* and *Funambulus palmarum*. Young specimens were consequently identified as having open or partially open interfrontal, interparietal, and exoccipital sutures. Adult cranial size differed between species, but no form of size correction (e.g., to unit size) was performed on measurements used to construct trajectories, therefore individual species' allometric trajectory estimates included size. Because ontogenetic material can rarely be perfectly age-/stage-sampled for non-model species, which include all those herein examined, the resultant trajectory estimates may be affected by what constitutes as sampling of a species' ontogeny. I used a similar sampling strategy to the study of Wilson and Sánchez-Villagra (2010), whereby the range in skull length between the smallest juvenile and largest adult reflected a range in size of at least 40%, that is, the smallest specimen had a cranial length of not more than 60% of the largest adult (Table S2). This number was originally based upon preliminary investigation of museum specimen availability for muroid and hystricognath species, and for the pygmy squirrel *Exilisciurus exilis,* the variance was slightly less (34.5%), although within the range of a previous study of ontogenetic allometry in a sciurid species (Cardini and Thorington 2006, Table 4). To examine the effect of differential ontogenetic sampling between clades, measurements for Ctenohystrica and muroids were subsampled, so that for each species, a trajectory was re-calculated based on specimens representing a range in cranial size of 35%, the lowest value for sciurids. The subsampled coefficients for muroids and Ctenohystrica were re-examined against sciurids, and showed small deviations from previous average estimates (see Fig. S1), hence no overall change to conclusions.

**Table 1:** List of species used in analyses. Notable extremes in body size are denoted as P – pygmy, G – giant. Tribe and clade membership (in parentheses) are based on the most comprehensive molecular phylogenetic framework for squirrels (Mercer and Roth 2003)

Clade	Species	*N*	Habit	Average body mass (g)
Pteromyini (V)	*H. lepidus*	27	Flying	89
Pteromyini (V)	*I. horsfieldii*	27	Flying	176
Pteromyini (V)	*P. leucogenys*	45	Flying (G)	2250
Pteromyini (V)	*P. vordermanni*	16	Flying (P)	100
Callosciurinae (III)	*L. insignis*	32	Ground	125
Callosciurinae (III)	*M. berdmorei*	40	Ground	195
Marmotini (IV)	*T. sibiricus*	23	Ground	85
Callosciurinae (III)	*C. notatus*	34	Tree	225
Callosciurinae (III)	*D. rufigenis*	28	Tree	240
Protoxerini (III)	*F. palmarum*	20	Tree	37
Protoxerini (IV)	*P. poensis*	17	Tree	114
Callosciurinae (III)	*R. laticaudatus*	32	Tree	221
Sciurini (V)	*S. vulgaris*	20	Tree	393
Callosciurinae (III)	*S. hippurus*	47	Tree	359
Callosciurinae (III)	*T. mcclellandii*	37	Tree	63
Ratufini (II)	*R. bicolor*	35	Tree (G)	1750
Callosciurinae (III)	*E. rufigenis*	27	Tree (P)	25

**Figure 2:**
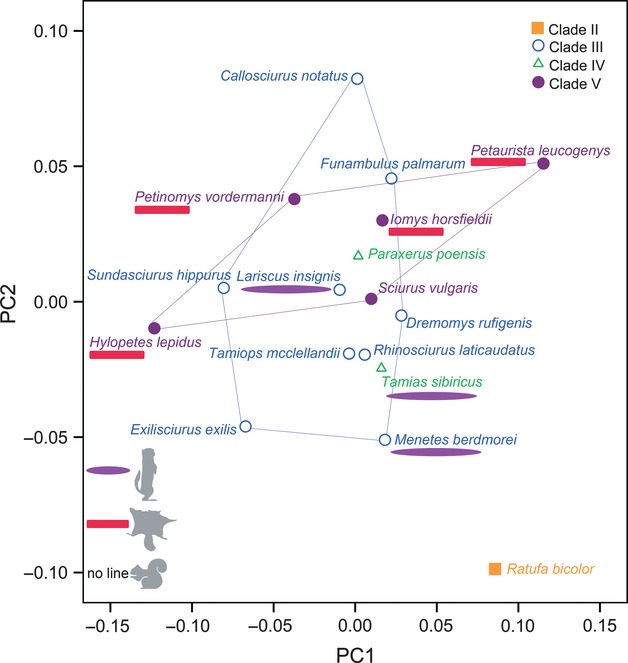
Allometric space for 17 squirrel species, comprising representatives from four (Clade II to Clade V) of the five major lineages denoted by Mercer and Roth (2003, Fig. 1). Each species is represented by a single point in allometric space, describing its allometric trajectory. Following Mercer and Roth (2003), Clade II (filled square) is monotypic, Clade III (open circle) comprises the Indo-Malayan tree squirrels (now grouped as Callosciurinae), Clade IV (open triangle) includes the Holarctic Marmotini and African and Central Asian Xerini in addition to nearly all tree squirrels from Africa, Clade V (filled circle) includes flying squirrels, all New World and some Old World tree squirrels. Habit groupings are denoted in the bottom left corner: flying (dashed solid line), ground (solid line), and tree (no line). PC1 = 25.5% and PC2 = 15.2% of sample variance.

**Figure 3:**
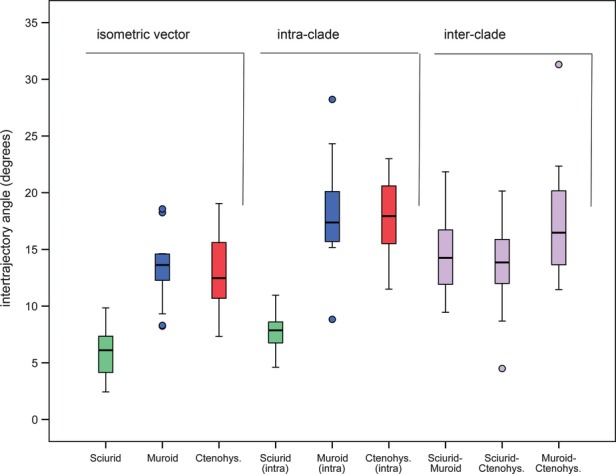
Boxplots showing the distribution of inter-trajectory angle comparisons (whiskers = minima and maxima excluding outliers, filled circles = outliers, filled horizontal bar = median value, boxes = middle two quartiles). Comparisons, from left to right, are between members of a clade and the isometric vector (coefficient value of 0.2425), within members of a clade, and between members of different clades. Clades and comparisons are denoted on the *x*-axis (Ctenohys. = Ctenohystrica).

Based on ontogenetic material available in collections, representatives were selected from four of the five major clades identified by Mercer and Roth (2003, Fig. 2). These comprised: (1) a monotypic clade containing *Ratufa* (Clade II, Fig. 2); (2) a major lineage containing other Indo-Malayan tree squirrels, now grouped as Callosciurinae (Clade III, Fig. 2); (3) a major lineage containing the Holarctic Marmotini and African and Central Asian Xerini as well as nearly all the tree squirrels from Africa (Clade IV, Fig. 2); and (4) a major lineage including the flying squirrels, all New World and some Old World tree squirrels (Clade V, Fig. 2). Species were chosen to encapsulate a diversity of habits (flying, tree, and ground) and included pygmy (*Hylopetes lepidus*, *Petinomys vordermanni*) and giant (*Petaurista leucogenys*) flying squirrels, in addition to pygmy (*Exilisciurus exilis*) and giant (*Ratufa bicolor*) tree squirrels. Typical body mass for each of the species measured herein was calculated from the literature (Table 1) and ranged from 25 g (*Exilisciurus exilis*) to 2.25 kg (*Ratufa bicolor*): for the majority of species, Nowak (1999) was taken as reference and, in addition, body mass data were taken from those compiled in a comprehensive review by Hayssen (2008).

### Morphometric analyses

All raw measurement data were log-transformed prior to further analysis. For each of the 17 species examined, a covariance matrix was generated and subjected to Principal Component Analysis (PCA).

Following the multivariate generalization of simple allometry (Jolicoeur 1963), the vector of first principal component coefficients (pc1: hereafter, lowercase letters denote principal components extracted from measurements of individual species) details the multivariate pattern of allometric growth. A new data set was created using the pc1 vectors from each individual PCA (Table S2). A second PCA was conducted using these vectors (i.e., individual species trajectories) as observations to produce an allometric space in which each point represented the allometric trajectory of a species (Klingenberg and Froese 1991; Gerber et al. 2008; Wilson and Sánchez-Villagra 2010). Allometric space is thus defined by the whole data matrix of *n* taxa × *p* allometric coefficients. Allometric space for squirrel species was ordinated using both phylogenetic groupings and habit groupings (flying, ground, tree). Habit groupings were examined because flying squirrels, in particular, display anatomical differences associated with a nocturnal lifestyle, and these include cranial modifications such as comparatively increased orbital size (Nowak 1999; Thorington and Santana 2007). Habit could be unambiguously assigned to each of the species under study using documented anatomical features of the cranium and postcranium, which distinguish flying, ground, and tree forms (Simpson 1945; Thorington et al. 1997; Nowak 1999).

To compare the patterning of allometric space for sciurids with that previously documented for the other two major clades of rodents (muroids and Ctenohystrica), a second analysis was conducted in which the first principal component (pc1) vectors generated in this work were combined with those of Wilson and Sánchez-Villagra (2010). The latter study used the exact same measurements and methods that are documented above. The combined data set represents allometric trajectories for 51 species, with equal sampling within each group (*N* = 17). A second analysis was conducted using this combined data set to, therefore, produce a plot of allometric space with 51 data points (species' trajectories), comprising cranial measurements from a total of 1620 specimens.

### Measures of allometric disparity

Several measures of disparity (=quantification of anatomical variability in a group of organisms, e.g., Zelditch et al. 2004) were used to quantify the magnitude of variance in allometric trajectories, referred hereafter as “allometric disparity”, both within sciurids and comparatively across representatives of all three major rodent clades.

To measure the amount of divergence between allometric trajectories, inter-trajectory angles were computed between all species within the sciurid clade and also between each sciurid species and each of the 34 species of muroids and Ctenohystrica examined by Wilson and Sánchez-Villagra (2010). To calculate the angle between the trajectories of two different species, the arc cosine of the dot product of the two vectors (pc1s) was used (Klingenberg 1996). In addition, each allometric trajectory was compared with the isometry vector, thereby providing a measure of distance to isometry (e.g., Jolicoeur 1963; Klingenberg 1998). In allometric space, isometry represents a fixed location, defined as a vector of length *p* with coefficients equal to *p*^−1/2^ whereby *p* is the number of variables (herein, isometric vector = 0.2425). Angles between species' pc1s and the isometric vector were also calculated using the same comparisons as for the inter-trajectory angles described above.

To compare allometric disparity between each of the three major clades, total variance (sum of univariate variances) was computed for each group of 17 allometric vectors separately. The total variance metric measures the spacing of species in allometric space, and is computed as the trace of the covariance matrix of allometric patterns, and also indirectly reflects the degree of parallelism of the trajectories (Gerber et al. 2008).

### Measures of adult disparity

For each of the three clades, adult disparities (=measures of variability in adult form) were estimated to compare with allometric disparity measures. Herein, allometric disparities are used to provide an estimate of allometric trajectory divergence within clades; however, comparatively disparate filling of allometric space may not necessary lead to comparatively greater disparity in adult morphospace. Disparately filled allometric space, indicating considerable variability of allometric trajectories, may lead to reduced adult morphospace filling as a consequence of reduced adult size variation among species. In reverse, when allometric trajectory patterning is conserved, greater adult disparities may be achieved by increased variation in size in a clade.

To assess how adult morphospaces are filled compared to their allometric counterparts, three measures of adult disparity were calculated for each clade. These were adult size disparity (using body mass data), adult shape disparity (using size-corrected measurement data), and adult size + shape disparity (using uncorrected measurement data). For each clade, data matrices of log cranial measurements were pruned to leave only adult specimens and for each species, an average adult morphology (set of measurements) was computed; therefore, three matrices each containing 17 adult species were created.

To calculate adult shape disparity, the effects of size were removed from the data matrices first. Burnaby's size correction method (Burnaby 1966) was used to project each matrix of log-transformed measurements onto the isometric size vector. The resultant size-corrected data are coordinates of the projected points, expressed in the coordinate system of the original variables (Klingenberg 1996). PCA was performed on each size-corrected matrix and in each case, scores were retained for all components extracted following the broken-stick model (Jackson 1993). The sum of variances for significant principal component axes was used to quantify adult shape disparity for each clade. Adult size + shape disparity was calculated also as the variance of principal component axes, but the raw adult measurements were inputted for the PCA (i.e., size correction was not first performed). In both cases, variance was calculated from the trace (sum of the diagonal elements) of the variance–covariance matrix of principal component scores (Zelditch et al. 2004). For each clade, the range in body mass among species was used as an estimate of adult size disparity. Besides the average body mass data for sciruids detailed in Table 1, data for muroids and Ctenohystrica were taken from Nowak (1999) (see Table S1).

### Statistical evaluation of trajectories and angles

The bootstrap approach was used to evaluate the stability of the allometric trajectory estimate of each species (pc1) (Efron and Tibshirani 1986). The method was used to generate standard error values, calculated as the standard deviation of the bootstrap distribution of each coefficient within an allometric trajectory (e.g., Klingenberg and Froese 1991; Klingenberg and Spence 1993). Samples were drawn with replacement 1000 times for each species.

The Kruskal–Wallis test, a non-parametric equivalent of ANOVA, was used to compare the medians of inter-trajectory angle values between clades and for each clade in relation to the isometric vector. Kruskal–Wallis tests were coupled with Mann–Whitney post-hoc pairwise tests to evaluate the statistical significance of comparisons. The resulting *P* values were further corrected using the Bonferroni method, as a conservative approach for multiple testing (Zar 1974).

The bootstrap approach was also applied to evaluate the measures of total variance that were generated separately for each clade. The total variance measure for each clade is based on the trace of the covariance matrix of 17 species, hence per clade, the within-species matrices were resampled 1000 times and their corresponding pc1s were generated. For each set of 17 species, these pc1s were compiled and used to generate 1000 covariance matrices. The trace of each of the 1000 covariance matrices was computed and then a confidence interval (CI) was calculated based on the 2.5% and 97.5% percentiles. Three sets of confidence intervals were therefore generated – one each for muroids, Ctenohystrica, and sciurids.

To test the null hypothesis that the allometric trajectories for each clade represent a sample from the same distribution, a permutation test was performed. The total group of trajectories (*N* = 51) were resampled without replacement and covariance matrices (17 × 17) were computed from the resampled trajectories. Original covariance matrices for muroids, Ctenohystrica, and sciurids were not deemed significantly different from one another if more than 5% of the test (*t*) statistic values calculated from permutation replicates were equal to or exceeded the observed *t* statistic value, which was calculated from mean and variance values.

All resampling methods were conducted using the Monte Carlo analysis and resample tools in PopTools (Hood 2010).

### Dietary habit

With the exception of *Rhinosciurus laticaudatus*, all species of squirrel were assigned to one of four dietary categories created by Wilson and Sánchez-Villagra (2010). Categories were based on food materials that were primarily incorporated into the diet: herbivore resistant (hr), herbivore soft (hs), omnivore soft (os), or omnivore resistant (or). The categories hr and or were adapted from the study of Samuels (2009) and the term “resistant” rather than “hard” (as per Wilson and Sánchez-Villagra 2010) was used to indicate that, herein, both hard food and tough food plant materials were grouped together. Studies of dietary morphology typically distinguish hard foods as those that are difficult to crack or break (stress-limitation), and tough foods as those that are difficult to detach in a piece to eat (displacement-limited) (see Lucas et al. 2000). The categories are defined as: hr – diet comprised primarily of plant matter including large quantities of fibrous plants (e.g., grass, bark, roots, and tubers) or dust and grit; hs – diet consisting mainly of plant matter that includes mostly soft leaves, fruits or seeds, and very little tough plant matter or dust/grit. The os and or categories reflect the same plant matter distinctions, but with the inclusion of animal matter, such as small eggs, insects, and worms. Rodents are opportunistic feeders, meaning that under circumstances such as resource shortage, switching from one diet to another is not uncommon; therefore it is not assumed that any species is obligated to a particular diet and the categories should not be considered in complete distinction from one another. *Rhinosciurus laticaudatus* was excluded from an a priori grouping, instead being ordinated as an ungrouped case, because it was the only species with an insectivorous diet, feeding strictly on large ants, termites, beetles, and earthworms (Nowak 1999). Principal component coefficients derived from the analysis of allometric trajectories of 50 species (hr = 18, hs = 8, os = 6, or = 18), represented by 1588 specimens, were used as input for canonical variates analysis (CVA).

## Results

### Allometric space for sciurids

Bootstrap results revealed that confidence intervals for allometric coefficients of sciurid trajectories included or were close to isometry (Table S2). The first two principal component (PC: hereafter, upper case letters denote the axes of allometric space, representing variation among species' allometric trajectories) axes of allometric space accounted for only 40.7% of variance and an additional 33.8% of variance was spread across PC3–PC6. These latter axes were plotted and comprised values of variance ranging from 13.6% down to 8.7% (Fig. S2).

### Allometric disparity metrics for sciurids

The average angle between allometric trajectories among squirrels was 8.9°. Inter-trajectory angle values ranged from 3.7° between *Lariscus insignus* and *Dremomys rufigenis*, to 14.9 degrees between the two flying squirrels *Hylopetes lepidus* and *Petuarista leucogenys* (Table S3). The average angle among species belonging to the Indo-Malayan lineage (Clade III) was 8.6° and, similarly, 8.7° among species belonging to Clade IV. Likewise, among ground (7.2°), tree (9.0°), and flying (10.7°) forms, values were broadly similar. Comparisons between species allometric trajectories and the isometric vector revealed an average angle of 5.8° (Table S4) (Fig. 3). The allometric trajectory for *Dremomys rufigenis* aligned most closely to the isometric vector (2.4°), whereas *Hylopetes lepidus* had the widest angle from the isometric vector (9.8°). Total variance among allometric trajectories for squirrels was 0.01238 (C.I. 0.0119–0.022).

### Allometric space for all rodents

**Figure 4:**
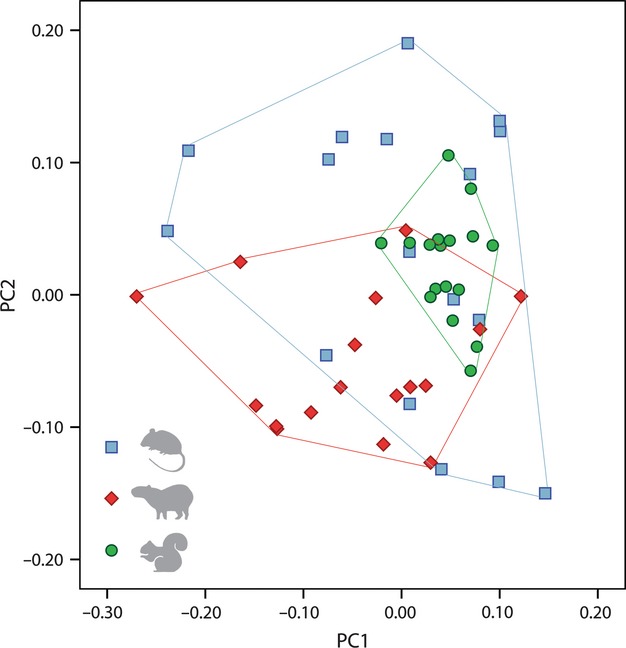
Allometric space for 51 rodent species, combining data of muroids (filled square) and hystricognaths (filled diamond) from Wilson and Sánchez-Villagra (2010) with those collected in this study for sciurids (filled circle). PC1 = 25.5% and PC2 = 15.2% of sample variance. Additional ordinations of PC1 versus PC3, PC4, PC5, and PC6 can be found in the online supplementary Figure S2.

Allometric space was constructed from 51 allometric trajectories, comprising equal numbers of representative species from muroids, Ctenohystrica, and sciurids (Fig. 4). The first two principal component axes represented 40.5% of the variance. Species with positive scores on PC1, which accounted for 23.0% of the sample variance, had a greater negative allometric coefficient for palatal width and basisphenoid length measurements compared with the mean growth trajectory. Change along PC2, accounting for 17.5% of the variance, reflected deviations from the mean trajectory in growth trends for nasal and premaxilla length measurements, as well as for length of the parietal. Sciurid allometric trajectories occupied a reduced range of morphospace, unlike those for the muroid and Ctenohystrica clades, which both had broadly overlapping and comparatively more disparate species ranges. In the case of sciurids, most species had small positive PC1 and PC2 coefficients (see Fig. 4).

### Allometric disparity metrics for all rodents

The average angle between all species trajectories was 14.1°. The closest pair of trajectories was *Thryonomys swinderianus* and *Sundasciurus hippurus* (4.4°) and the widest inter-trajectory angle was 25.1° between *Petaurista leucogenys* and *Cavia porcellus* (Table S5). When examining all inter-trajectory angles, the majority of comparisons (84%) between Ctenohystrica or muroid species and sciurids resulted in a value that exceeded 10.7°, the greatest difference between two sciurid trajectories (Fig. 3). Inter-trajectory angles were significantly smaller among sciurid species compared with those among muroids (χ^2^ = 232.2, *P* = <0.0001, Bonferroni corrected) and Ctenohystrica (χ^2^ = 232.2, *P* < 0.0001, Bonferroni corrected). The average angle between a sciurid and muroid was 14.5° and similarly 14.0° between a sciurid and a representative of Ctenohystrica (Fig. 3). A similar pattern was evident for angles between species and the isometric vector. The average angle to the isometric vector was significantly smaller among sciurids (5.8°) compared with among muroids (13.2°; χ^2^ = 30.66, *P* < 0.0001, Bonferroni corrected) and Ctenohystrica (13.0°; χ^2^ = 30.66, *P* < 0.0001, Bonferroni corrected) (Fig. 3). Among muroids, angles to the isometric vector ranged from 8.2° for *Tachyoryctes splendens* to 18.7° for *Rhizomys sumatrensis*. Similarly, among Ctenohystrica, angle values ranged from 7.3° for *Thryonomys swinderianus* to 19.0° for *Cavia porcellus* (Fig. 3). Total variance among allometric trajectories for all rodents examined was 0.0372. For separate analyses, total variances were 0.0409 (C.I. 0.039–0.0481) and 0.0499 (C.I. 0.0433–0.051) for muroids and Ctenohystrica, respectively. Permutation results indicated that measures of variance were significantly different between sciurids and both muroids (*P* < 0.001) and Ctenohystrica (*P* < 0.001), but not between muroids and Ctenohystrica (*P* = 0.57).

### Comparison of allometric trends for individual variables

**Figure 5:**
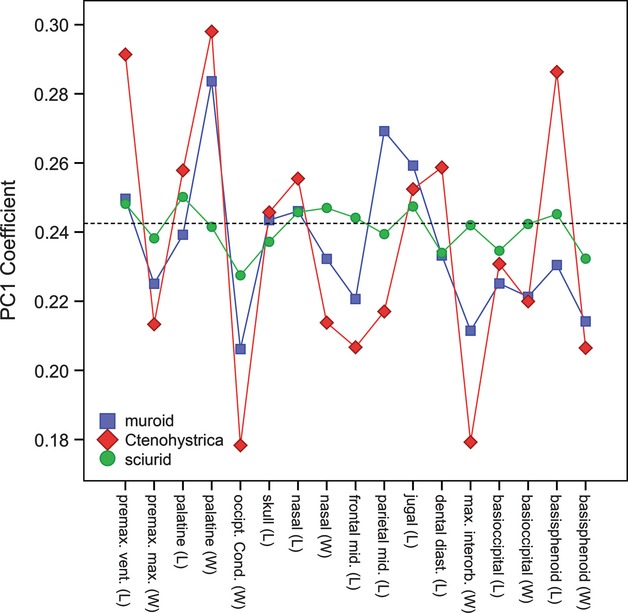
Average pc1 coefficient (allometric vector) for each variable measured. Averages are computed for each clade separately (muroid – filled square, Ctenohystrica – filled diamond, sciurid – filled circle) based on pc1 coefficient values of 17 representative species. The dashed line indicates a value of 0.242, which refers to the length of the isometric vector in multivariate space, defined by the number of variables (*p*) measured (length = *p*^−0.5^).

Comparison of allometric coefficients across muroids, Ctenohystrica, and sciurids on a variable-by-variable basis revealed a broad similarity between absolute coefficient values for muroids and hystricognaths in most cases, whereas sciurids commonly departed from the other two clades (Fig. 5). For most variables, allometric coefficients were close to isometry (dotted line, Fig. 5) for sciurids. The greatest discrepancy in growth trends between the clades was for maximum interorbital width (range = 0.063) and palatine width (range = 0.057). In all clades, skull length was least variable and closest to isometry (range = 0.008) (Fig. 5).

### Canonical variates analysis using dietary habit

**Figure 6:**
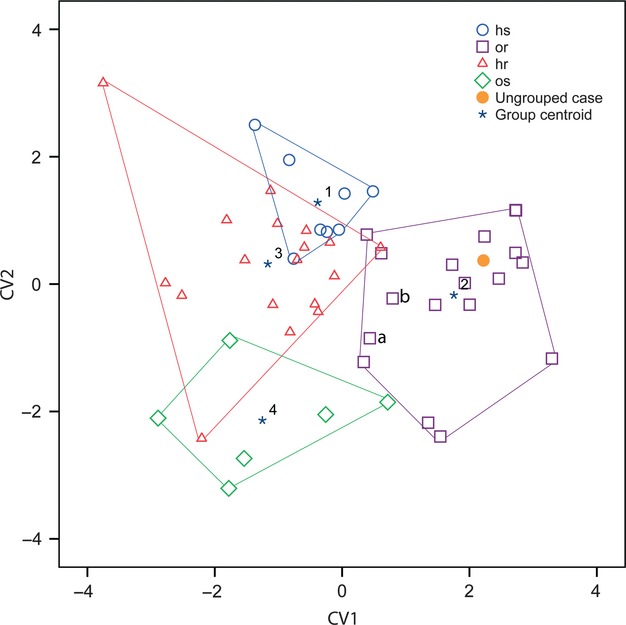
Canonical variate analysis of principal component scores representing 95% of variance in allometric space computed for 51 rodent species. A priori groupings constructed for dietary habits: hs – herbivore soft (open circle), os – omnivore soft (open diamond), hr – herbivore resistant (open triangle), or – omnivore resistant (open square). The insectivorous sciurid *Rhinosciurus laticaudatus* (filled circle) was ordinated as an ungrouped case. The two non-sciurid members of the or category are identified as *Capromys pilorides* (a) and *Atherurus africanus* (b). See Methods for details of dietary grouping criteria.

Of 50 species, 41 (82%) were correctly classified into one of the four *a priori* defined dietary categories, although classification success fell with cross-validation to 70% (Table S6). The first canonical variate (CV1) accounted for 59.8% of the variance (eigenvalue = 1.97) and separated the herbivores eating resistant material, typically having negative values along that axis, from omnivores eating resistant material (Fig. 6). The second canonical variate (CV2) accounted for 28.4% of the variance (eigenvalue = 0.93) and separated herbivores eating soft foods from omnivores eating soft foods. All of the sciurids examined herein were classified as having an omnivorous resistant diet (*n* = 16), which substantially increased the sampling of species within that category, previously represented by two hystricognath species in the analyses of Wilson and Sánchez-Villagra (2010). It is not possible, though, to conclusively rule out a phylogenetic influence on groupings because of the high number of sciurids in the omnivorous-resistant category. If phylogenetic affinity was of greater influence than dietary habit, one may expect the two non-sciurids (*Capromys pilorides* and *Atherurus africanus*) within the omnivore-resistant category to be located away from, or at the extreme edges of the sciurid spacing for that group; however, this does not appear to be the case in Figure 6 (see points a and b). Nevertheless, *Rhinosciurus laticaudatus*, the ungrouped case (insectivore), also nested closely within the omnivore-resistant grouping, containing the other sciurids. The inclusion of additional species to the omnivore-resistant category of Wilson and Sánchez-Villagra led to slight overlap along CV1 with the herbivore-resistant category, namely *Rhizomys sumatrensis* (hr) sharing similar growth patterns to *Exilisciurus exilis* (or) and *Petinomys vordermanni* (or), whereas the other two groups (hs and os) remained completely distinct.

### Adult disparity metrics

The greatest overall differences in adult disparities between clades were for measures of size, calculated from ranges of average body mass. For sciurids in this sample, adult size disparity was 1.725, which was less than for muroids (18.47), and both clades differed from 66.43, the value for Ctenohystrica. Measures of adult shape and adult size + shape disparities were slightly greater for Ctenohystrica (shape = 0.1083 [C.I. 0.092–0.125], size + shape = 0.6846 [C.I. 0.612–0.725]) than for muroids (shape = 0.1028 [C.I. 0.088–0.119], size + shape = 0.6744 [C.I. 0.611–0.733]), and both clades had much higher disparities than sciurids (shape = 0.0312 [C.I. 0.0289–0.0366], size + shape = 0.2729 [C.I. 0.264–0.301]).

## Discussion

Allometry has widely been regarded as a constraint that channels variation in fixed directions of phenotypic space, based on interpretations of empirical evidence across a range of intra- and interspecific comparisons (e.g., Gould 1974; Ross 1995; Abdala et al. 2001; Flores et al. 2003). Several recent studies that have connected evolution and development through the application of morphometric methods to extract quantitative descriptions of macroevolutionary allometry serve to illustrate that allometric trajectories themselves evolve (Gerber et al. 2008; Adams and Nistri 2010; Klingenberg 2010b; Wilson and Sánchez-Villagra 2010, 2011). In this study, I examined the evolution of allometric trajectories among three clades of rodents to determine the extent of covariance structure changes and to test the temporal persistence of an adaptive ecological model for allometric evolution.

The results show that in comparison to Ctenohystrica and muroids, sciurids differed significantly in terms of allometric evolution trends. First, their occupation of allometric space was comparatively less spread than that of the other two major clades (Fig. 4), further evidenced by significantly smaller values for measures of total variance among sciurid allometric trajectories, and comparatively smaller inter-trajectory angles between members of the clade (Tables S3–S5). Second, comparisons of angles between trajectories and the isometric vector showed that sciurid trajectories tended to be positioned much closer to isometry in allometric space than did those of either muroids or Ctenohystrica (see also Fig. 5).

Therefore, based on the species examined herein, and assuming sciurids as sister to the other two clades, allometric evolution in rodents appears to be characterized by a comparatively reduced magnitude of inter-trajectory change and allometric growth patterns in sciurids that deviate little in terms of coefficient magnitude from isometry, in contrast to a greater magnitude of difference between trajectories and increased variation in growth patterns among both Ctenohystrica and muroids. Only crown-group rodents were examined, due to constraints of allometric data availability, hence a statement cannot be made about whether either pattern can be considered apomorphic for the “order”. Whether a shift happened, and if so from less to more trajectory variability, or vice versa, is a point that would require a phylogenetically explicit analysis, ideally incorporating additional groups on a scale similar to the sampling in this study. Relevant work currently being conducted in this direction will also involve optimizing allometric data onto the phylogeny of the herein examined species, to determine the precise nodes associated with, and the polarity of, key shifts in allometric coefficient values. Either way, rodents do not seem to follow the general trend of conserved covariation patterning that has been reported, based on adult traits, for several other mammalian groups, and has been suggested to be consequent of stabilizing selection (Hallgrímsson et al. 2009). A recent broad-scale comparison of covariance patterns across 15 mammalian “orders” indicated highly conserved covariance structure, and although each “order” was represented by only one or two species (Porto et al. 2009), the result was analogous to more detailed intra-clade comparisons of groups including neotropical marsupials, humans and other primates (Marroig and Cheverud 2001; González-Jose et al. 2004; Ackermann 2005; Shirai and Marroig 2010; Singh et al. 2012). Nevertheless, rodents are not the only group to display considerable variability in covariance structure, as Goswami (2007) indicated in her study on integration patterns in australodelphian marsupials. It is also not implausible that covariance patterns may alter over a short period of time, as has been shown for laboratory mice (Pavlicev et al. 2008; Hallgrímsson et al. 2009). In part, these differences may reflect the way in which covariance structure arises, that is through the variation in developmental processes that generate covariance (e.g., Hallgrímsson et al. 2009).

Unlike Ctenohystrica and muroids, sciurids have both low allometric disparity and low adult disparity values, which appears to suggest that conserved trajectory patterning has constrained adult disparity in the group. Because sciurids in the sample are the clade with the smallest adult size variation, the argument could be made that lower levels of size variation in the sample may simply explain the much lower allometric and adult disparity values, particularly as the estimates of allometric disparity do not include a representative of the genus *Marmota*, which are the largest members of the sciurid clade with an average body mass of around 5 kg (Nowak 1999). Although I cannot rule out that allometric disparity may be underestimated due to not sampling *Marmota*, I re-computed adult disparities to consider the potential limitation to the conclusion. If I include adult specimens of *Marmota marmota* and re-compute adult disparities, adult size disparity for the sciurid clade increases to 5.225 and both adult shape disparity (0.0414) and size + shape disparity (0.3580) increase compared with previous sciurid measures, but the latter two values are nevertheless not of a similar magnitude to the other clades, suggesting that adult morphospace for sciurids is less disparately filled. As acknowledged earlier, allometric disparity and adult disparity are not the same, and while it is reasonable to conclude that the inclusion of *Marmota* had a small effect on adult disparities, the same cannot be assumed for allometric disparities.

The results here are the first to show a broad-scale relative conservatism in covariance structure for sciurids and are consistent with the preliminary investigations made by Roth (1996), who proposed that the sciurid cranium displays subtle, continuous variation, and that the high level of integration found by Olson and Miller (1958) for *Sciurus niger* may be applicable on a more general level across the clade. Studies of the mandible have also documented isometric scaling for different sciurid species (Velhagen and Roth 1997; Swiderski 2003; Hautier et al. 2009; Swiderski and Zelditch 2010), and these are considered to be related to maintaining functional relationships such as mechanical advantages. The latter evidence, together with the results of recent biomechanical analyses in the sciurid cranium (Cox et al. 2012) that reveal a highly efficient morphology for resisting stresses associated with their gnawing behavior (durophagy), appears to support the fundamental role of ecology in generating conservatism of sciurid allometric trajectories.

Among rodents, squirrels are usually pigeon-holed for their skilled capabilities in processing resistant foodstuffs (e.g., Ball and Roth 1995; Roth 1996). Apart from the ungrouped (insectivorous) case of *Rhinosciurus laticaudatus*, all Sciuridae in the study sample were categorized as having an omnivorous resistant diet. *Rhinosciurus laticaudatus* nested among its sciurid relatives in CVA space (Fig. 6), all of which occupied the positive region of CV1. Both pygmy squirrels had low values along CV1 and were positioned near to the herbivore-resistant group, particularly close to *Rhizomys sumatrensis*, which was the only member of the latter group to have a positive score along CV1, resulting in slight overlap between the two groups. The species belonging to the herbivore-resistant category occupy a region of allometric space (for all 51 species) equating to a larger than average negative allometric coefficient for nasal and premaxilla width measures. The outcome of growth for these traits is a comparatively shorter rostral region, as also found among the pygmy squirrels here. Members of the herbivore-resistant grouping also typically grow to have a wider nasal and overall deeper skull that act to support larger masticatory muscles and mitigate stresses borne from processing hard and fibrous foods. These results are in line with other studies that have reported differences in adult cranial morphology related to dietary adaptation in sciurids (e.g., Cardini and O'Higgins 2005) and also other rodents (Michaux et al. 2007; Samuels 2009). Furthermore, among disparity analyses for other mammals, diet has also been shown to play an important role in determining shifts in cranial morphology. In particular, large-scale studies of carnivorous mammals have shown convergent morphology in relation to dietary habit, with evident differences among hypercarnivorous, omnivorous, and insectivorous forms (Wroe and Milne 2007; Goswami et al. 2011). In the study by Goswami et al. (2011), some phylogenetic structure was also evident in morphospace occupation and greater disparity likely reflected greater ecological diversity in some clades.

Examining the allometric space created for sciurids, similar to that previously constructed for muroids and Hystricomorpha (Wilson and Sánchez-Villagra 2010), overall a clear trend between taxon spacing and body mass (size) is not evident despite the repeated evolution of dwarf and giant forms that is known to characterize the group (e.g., Mercer and Roth 2003; Hautier et al. 2009). For instance, the study sample comprises pygmy forms (*Exilisciurus exilis* and *Petinomys vordermanni*) as well as giant forms (*Petuarista leucogenys* and *Ratufa bicolor*), and although these appear located at opposite regions of PC1 (Fig. 2), with smaller species occupying the negative region of that axis, their positions are shared with other taxa of different body mass. *Sundasciurus hippurus*, for example, is much larger than *P. vordermanni*, *Hylopetes lepidus,* and *E. exilis*, but also shares a similar score along PC1. In the case of pygmy squirrels, the studied species have broadly similar PC1 scores and differ along PC2, with the pygmy flying squirrel *P. vordermanni* grouping more closely with other flying squirrels that have a higher value along this axis, indicating a more negative than average allometric growth of the dental diastema, meaning achievement of slightly shorter rostral region. That the pygmy flying squirrel *P. vordermanni* groups more closely with other flying squirrels than with the dwarf tree squirrel *E. exilis* is perhaps not surprising based on the study of Roth (1996) who showed that flying squirrels exhibit morphological similarities irrespective of their size, namely an anterior displacement of the eyes and constricted orbits likely facilitating stereoscopic vision for gliding and landing. These features also result in a shortened rostral region, partly reflected in the negative allometric coefficients for nasal length among the majority of flying squirrels here (Table S2). Hautier et al. (2009) showed that pygmy flying squirrels have grossly similar mandibular morphology to other flying squirrels, and suggested that pygmy flying squirrels had a divergent static allometric trajectory compared with other pygmy squirrels. In the sample, giant squirrels are found to delimit the extreme range of variation on PC1, explained by faster than average widening of the basispheniod, narrowing of the palate, widening of the premaxilla, and shortening of the frontal. These features suggest convergence to a wider, shorter rostrum and compact mid-cranial region, coupled with comparatively large cranial dimensions in these sciurids.

## Conclusions

The quantification of allometric disparity over the course of rodent evolution in this study provides insights into allometric trajectory evolution for the largest mammalian “order”, focusing on patterns in the three major constituent lineages. The results indicate that Sciuridae have different patterns of allometric trajectory evolution compared with muroid and Ctenohystrica rodents. Sciurids possess a comparatively reduced magnitude of inter-trajectory change and allometric coefficients with small deviation from isometry, whereas a greater magnitude of difference between trajectories and increased variation in growth patterns is found for both Ctenohystrica and Muroidea. Common changes in covariance structure (=allometric trajectory variation) among Ctenohystrica and Muroidea resulted in higher values for all measures of adult disparity compared with Sciuridae, indicating that covariance structure modification, rather than conservatism, may result in increased adult morphological diversity. Generally compared with other mammalian clades, rodents appear different in their common use of changes in covariance structure and shifting strategies to fill adult morphospace. Further broad-scale outgroup sampling and explicit phylogenetic testing will help trace the potential polarity of the differences observed here between clades.

## Supporting Information

**Figure S1.** Results of subsampled pc1 coefficient (allometric vector) values for each variable measured.

**Figure S2.** Allometric space for 17 squirrel species, comprising representatives from four (Clade II to Clade V) of the five major lineages denoted by Mercer and Roth (2003, Fig. 1). Each species is represented by a single point in allometric space, describing its ontogenetic trajectory. Following Mercer and Roth (2003), Clade II (filled square) is monotypic, Clade Ill (open circle) comprises the lndo-Malayan tree squirrels (now grouped as Callosciurinae), Clade IV (open triangle) includes the Holarctic Marmotini and African and Central Asian Xerini in addition to nearly all tree squirrels from Africa, Clade V (filled circle) includes flying squirrels and most New World tree squirrels. Variance represented by axes: PCi = 25.5%, and PC3 = 13.6% (A), PC4 = 10.9% (B), PC5 = 9.6% (C), PC6 = 8.7% (D).

**Table S1.** Body mass data for Ctenohystrica and muroids.

**Table S2.** Allometric coefficients for sciurid species analyzed in this study.

**Table S3.** Angle measurements (degrees) between sciurid species.

**Table S4.** Angle measurements (degrees) between ontogenetic trajectories and the isometric vector.

**Table S5.** Matrix of angle measurements (degrees) between sciurid (x) and muroid and Ctenohystrica (y) species.

**Table S6.** Canonical Variates Analysis results.
