# Supplementary material for: Allometric disparity in rodent evolution
Source: Ecol Evol. 2013 Mar 5;3(4):971–84. doi: 10.1002/ece3.521 (PMC3631408; doi:10.1002/ece3.521)

Figure S1: Average pc1 coefficient (allometric vector) for each variable measured. Muroid and Ctenohystrica values have been computed from subsampled data sets that represent the lowest range in cranial size (35% difference in sciurids), to examine the effect of differences in ontogenetic sampling between clades. Refer to Fig. 5 for comparison. The dashed line indicates a value of 0.242, which refers to the length of the isometric vector in multivariate space, defined by the number of variables (p) measured (length = p^-0.5).

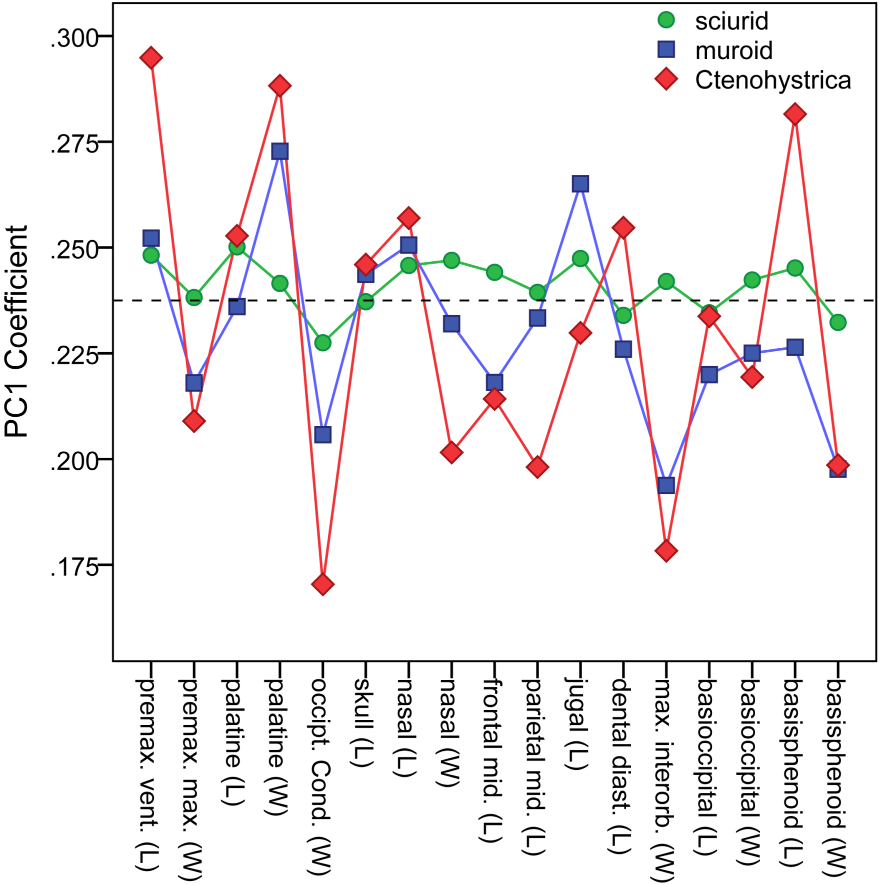

Supplement: Supplementary file 1 [file ece30003-0971-SD1.pdf]
