# Supplementary material for: Allometric disparity in rodent evolution
Source: Ecol Evol. 2013 Mar 5;3(4):971–84. doi: 10.1002/ece3.521 (PMC3631408; doi:10.1002/ece3.521)

Figure S2: Allometric space for 17 squirrel species, comprising representatives from four (Clade II to Clade V) of the five major lineages denoted by Mercer and Roth (2003, Figure 1). Each species is represented by a single point in allometric space, describing its ontogenetic trajectory. Following Mercer and Roth (2003), Clade II (filled square) is monotypic, Clade III (open circle) comprises the Indo-Malayan tree squirrels (now grouped as Callosciurinae), Clade IV (open triangle) includes the Holarctic Marmotini and African and Central Asian Xerini in addition to all tree squirrels from Africa, Clade V (filled circle) includes flying squirrels and most New World tree squirrels. Variance represented by axes: PC1 = 25.5%, and PC3 = 13.6% (A), PC4 = 10.9% (B), PC5 = 9.6% (C), PC6 = 8.7% (D).

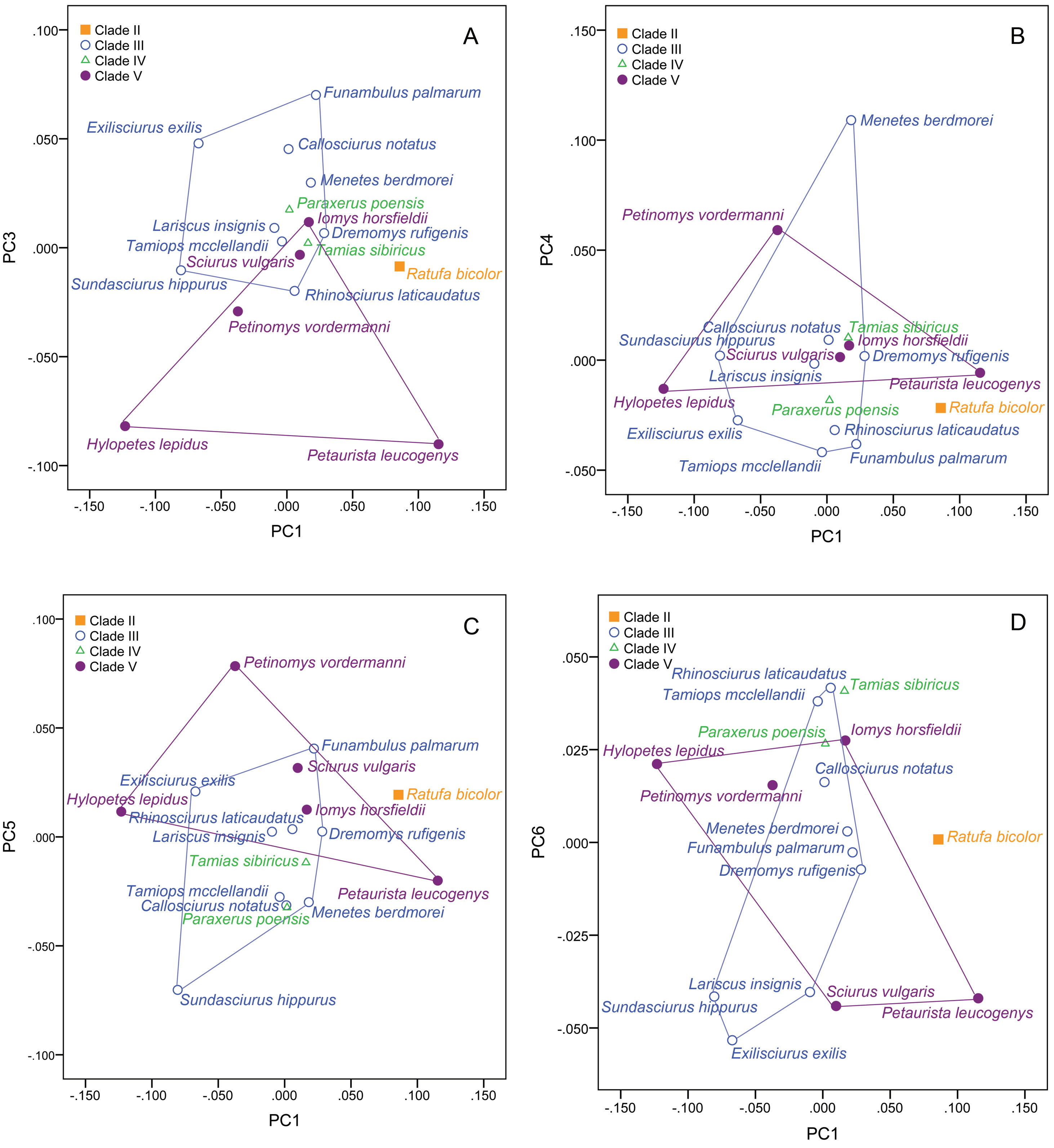

Supplement: Supplementary file 2 [file ece30003-0971-SD2.pdf]
